# Exploring Bone Health in Cystic Fibrosis: A Study From a Lung Transplantation Center and Strategy for Clinical Care

**DOI:** 10.1002/ppul.71210

**Published:** 2025-07-16

**Authors:** Gökçen Kartal Öztürk, Ece Halis, Ece Ocak, Aykut Eşki, Damla Gökşen, Samim Özen, Fevziye Çoksüer, Esen Demir, Figen Gülen

**Affiliations:** ^1^ Department of Pediatric Pulmonology Ege University Faculty of Medicine İzmir Turkey; ^2^ Department of Pediatric Pulmonology, Tepecik Education and Research Hospital University of Health Sciences İzmir Türkiye; ^3^ Department of Pediatric Endocrinology Ege University Faculty of Medicine İzmir Turkey

**Keywords:** bone disease, bone mineral density, children, cystic fibrosis, lung transplantation

## Abstract

**Background:**

Cystic Fibrosis Bone Disease (CFBD) is a known complication in children with CF and may cause serious problems in adulthood or transplantation processes. This study aimed to identify potential predictable risk factors for the development of low BMD by evaluating pediatric patients screened with DXA as a “Heart‐Lung Transplantation Center” and created new strategic plans to improve our CFBD screening program by evaluating our results in literature and guidelines recommendations.

**Methods:**

This retrospective cohort study includes 86 children ages 6–18 years with CF who underwent at least one DXA scan between August 2016 and October 2024. Participants were compared according to BMD *z* scores and the relationship between BMD and disease‐related parameters was evaluated.

**Results:**

The rate of DXA screening in our center was 81.1% over 8 years of age and 72.8% over 6 years of age. 41.8% of our population had abnormal BMD (*z* scores < −1), and the rate of very low BMD (*z* scores < −2) was 17.4%. The frequency of abnormal BMD was higher in children with BMI< 50th percentile, SKS ≤ 70, low FEV_1_ *z* score, respiratory microorganism colonization, ≥ 2 annual pulmonary exacerbations, required respiratory support, low albumin, and high CRP levels. Systemic inflammation marker CRP increase was the most predictable parameter for low BMD.

**Conclusion:**

This study informs clinical practice by highlighting the need for multidisciplinary interventions, such as earlier evaluation of DXA scans due to the risk factors and poor clinical conditions, a consistent follow‐up protocol, individualized nutrition programs with the dietitian, and enhanced physical therapy.

## 1 Introduction

Cystic Fibrosis Bone Disease (CFBD) is a known complication in children with CF, but the pathophysiology of the disease has not yet been completely understood. Known contributing factors of low bone mineral density (BMD) include malnutrition, CF transmembrane conductance regulator (CFTR) protein dysfunction, calcium, vitamin D, and vitamin K deficiency, delayed puberty and hypogonadism, decreased physical activity, exogenous glucocorticoids, respiratory infections and systemic inflammation, and CF‐related diabetes (CFRD) and liver disease [1, 2, 3]. The loss of BMD in CF is associated with recurrent pulmonary infection and reduced pulmonary function. Moreover, the development of CFBD causes poor clinical conditions, especially with increasing age [4]. In some lung transplantation (LTx) centers, low BMD is a contraindication to Tx. Pre‐transplant lower BMD and pulmonary function are associated with the risk of developing fractures after Tx [5]. Furthermore, a rapid decline in BMD can be observed in approximately 73% of transplant recipients, while the risk of nonvertebral fractures is increased two‐fold for men ages 25–45 years and women ages 16–34 years compared to the general population [6].

In pediatric patients, limited information is available about the clinical outcomes of CFBD. Low BMD in CF patients is likely to occur in childhood or adolescence, so with increasing life expectancy, preventing these childhood‐onset complications is important. Therefore, the European CF consensus recommends that all patients aged 8–10 years or older with CF undergo dual‐energy X‐ray absorptiometry (DXA) scan screening to monitor bone health [7]. However, despite these recommendations adherence is variable and inadequate in many centers. The screening rate is about 66% even in the adult population [8].

In this retrospective study, we aimed to examine our current status as a “Heart‐Lung Transplant Department” in CFBD and identify potential predictable risk factors for the development of low BMD.

## 2 Materials and Methods

### 2.1 Study Design and Population

This retrospective cohort study evaluates children ages 6–18 years with CF at Ege University Department of Pediatric Pulmonology. Children who underwent at least one DXA scan between August 2016 and October 2024 were included in the study. Patients with a lack of data in many clinical measurements and without regular follow‐up and DXA scans were excluded from the study. The Local Research Ethics Committee approved the study (20‐6 T/56) and all subjects provided written informed consent.

Clinical measurements within 3 months of the DXA scan in stable clinical condition were recorded from our patient data recording system. Pretreatment results were used for patients with modulatory therapy. Participants were compared according to BMD *z* scores and the relationship between BMD and disease‐related parameters was evaluated.

### 2.2 Bone Mineral Densitometer

The BMD of the lumbar spine was determined by DXA using a Hologic Horizon WI densitometer and was expressed as *z* scores. The last scan was used for subjects with more than one screening. BMD *z* score was adjusted for height and defined as normal for *z* scores greater than −1, moderately low for between −1 and −2, and very low for less than −2 [3].

### 2.3 Clinical Measurements

Demographic and clinical data included age, gender, anthropometric measurements, clinical score (Shwachman–Kulczycki score), genotype, nutritional status (gastrostomy tube, vitamin/mineral supplementation), CF‐related conditions (pancreatic sufficiency, liver and renal diseases, diabetes), history of fracture, medical treatment (systemic steroid, inhaled steroid, acid suppression) at the time of the DXA scan, and the number of annual pulmonary exacerbations and the presence of respiratory microorganism colonization the previous year before the scan.

Body mass index (BMI) was calculated with the weight (kilograms)/height (m2) formula and SD scores were according to the percentile curve for Turkish children [9]. CF genotypes were grouped as delta F508 mutation (homozygous or heterozygous) and other mutations. The Shwachman–Kulczycki score (SKS) was calculated by scoring the nutritional status, physical examination, chest X‐ray findings, and general activity. Overall, 86–100 points are excellent, 71–85 good, 56–70 mild, 41–55 moderate, and ≤ 40 severe [10]. Pancreatic exocrine insufficiency was defined with low fecal elastase and the use of pancreatic enzymes, and the presence of microorganism colonization was positive as respiratory cultures in at least half of the previous year's cultures [11]. Physical activity was defined as physical exercise exceeding 30 min per day and was grouped as no activity, 1–3 days, and > 3 days per week.

### 2.4 Laboratory Data

Biochemical data [fasting blood sugar (mg/dL), albumin (gr/L)], systemic inflammation markers [c‐reactive protein (CRP, mg/L), immunoglobulin G (IgG, mg/dL)] and bone metabolism‐related markers [calcium (mg/dL), phosphorus (mg/dL), magnesium (mg/dL), alkaline phosphate (U/L), vitamin D level (ng/mL), parathyroid hormone (ng/L), osteocalcin (µg/L)] were recorded from clinically stable conditions within 3 months of the DXA scan.

### 2.5 Pulmonary Function Test

The pulmonary function test (PFT) was performed by spirometry (Flowhandy ZAN 100, Germany) following the American Thoracic Society standards by measuring forced expiratory volume in 1 s (FEV_1_), forced vital capacity (FVC), and forced expiratory flow during the middle half of FVC (FEF_25‐75_) [12]. Results are expressed as *z* scores calculated using the global lung function initiative (GLI) reference equations.

### 2.6 Statistical Method

Statistical analysis was performed using IBM SPSS Statistics 25.0 (IBM Corp, Armonk, NY, USA). The Kolmogorov‐Smirnov test was used to test the normal distribution of the numerical variables. The numerical variables were presented as median (minimum‐maximum) and mean (± standard deviation), and categorical variables were as percentages (%) and were analyzed using the Pearson χ2 test or Fisher's exact test. Normal and abnormal BMD groups were compared with two independent samples *t*‐tests and the Mann–Whitney *U* test. One‐way analysis of variance (ANOVA) and the Kruskal–Wallis test were used to compare groups with BMD *z* scores. Spearman's and Pearson's Correlation tests performed correlation analysis between continuous parameters. Logistic regression analysis was used to determine potential predictable risk factors of low BMD in patients. Statistical significance was accepted as *p* < 0.05.

## 3 Results

Medical records of 211 children with CF followed in our department during the study period were reviewed. Ninety three children with age criteria, five with a lack of clinical data, nine without regular follow‐up, and 18 without DXA scans were excluded.

**Figure 1:**
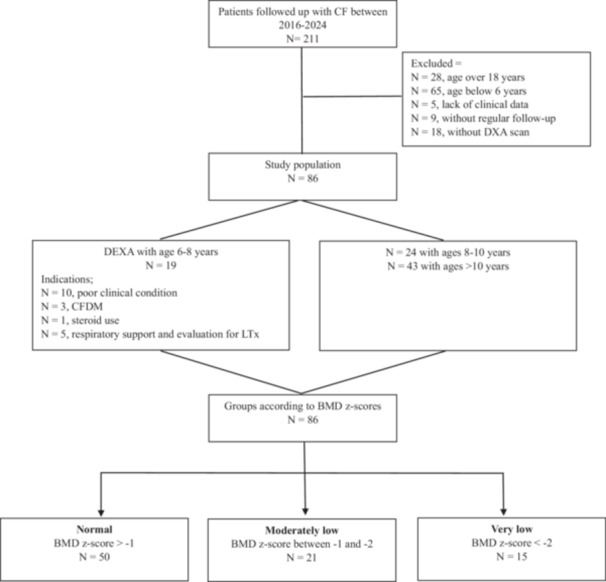
Flow chart of the study group.

The median age of the 86 children included in the study was 121 months, and 19 (22%) children were 6–8 years, 24 (27.9%) were 8–10 years, and 43 (50%) were over 10 years. Indications for DXA at 6–8 years of age were poor clinical condition in 10 children, CFDM in three, corticosteroid use in one, and respiratory support and evaluation for LTx in five (Figure 1). The rate of DXA screening was 72.8% over 6 years of age.

The study population was 52.3% (*N* = 45) female, the mean BMI *z* score was −0.96 ± 1.40, and 13.9% (*N* = 12) had F508del homozygous mutation. The mean BMD *z* score was −0.79 ± 1.25, and normal was at 58.1% (*N* = 50) of patients, and abnormal was 41.8% (*N* = 36) [moderately low at 24.4% (*N* = 21) and very low at 17.4% (*N* = 15)] (Table 1).

**Table 1:** Clinical characteristics of the study group.

	*N* = 86
Age, months (median, min‐max)	121 (72–216)
Gender, F/M	45/41
Body mass index (kg/m^2^), SD*	−0.96 ± 1.40
Mutations, (%)	
F508del homozygous	12 (13.9)
F508del heterozygous	32 (37.2)
Others	42 (48.8)
Colonization, (%)	60 (69.7)
Microorganism	
* Staphylococcus aureus*	33 (38.3)
* Pseudomonas aeruginosa*	39 (45.3)
Others	23 (26.7)
CF‐related disease, (%)	
Pancreatic insufficiency	80 (93)
Liver disease	11 (12.7)
Renal disease	—
Diabetes	12 (13.9)
Physical activity, (%)	
No activity	53 (61.6)
1–3 days/week	28 (32.6)
≥ 3 days/week	5 (5.8)
Pulmonary function tests*	
FEV_1,_ *z*‐score	−2.12 ± 2.25
%	78.07 ± 27.57
FVC, *z*‐score	−2.54 ± 2.16
%	71.23 ± 24.79
FEV_1_/FVC	0.59 ± 1.47
FEF_25‐75_	−0.88 ± 2.33
BMD, *z* score*	−0.79 ± 1.25
BMD, (%)	
Normal	50 (58.1)
Moderately low	21 (24.4)
Very low	15 (17.4)

Abbreviation: SD, standard deviation.

* mean ± SD.

### 3.1 Comparison of Children According to the BMD Group

#### 3.1.1 Clinical Characteristics

Age, gender, duration of illness, genotype, CF‐related diseases, nutrition status, and physical activities were similar between the groups (*p* > 0.05). In children with abnormal BMD, BMI *z* scores, SKS, FEV_1_, and FVC *z* scores, and albumin levels were lower, while the presence of respiratory microorganism colonization and number of annual pulmonary exacerbations, frequency of patients with respiratory support and evaluation of LTx, and CRP levels were higher compared to normal BMD (*p* < 0.05) (Table 2).

**Table 2:** Comparison of patients according to BMD groups.

	BMD > −1	BMD < −1				
	Normal (*N* = 50)	Total (*N* = 36)	BMD −1 and −2 Moderately low (*N* = 21)	BMD < −2 Very low (*N* = 15)	p^1^	p^2^
Age, months	133.8 ± 45.01	136.33 ± 47.06	134.09 ± 51.82	139.46 ± 41.01	0.80	0.91
Gender (F/M)	27/23	18/18	12/9	6/9	0.44	0.57
Duration of illness, months	90.42 ± 54.17	105.27 ± 50.48	108.66 ± 50.34	100.53 ± 52.05	0.20	0.39
BMI, SD	−0.61 ± 1.38	−1.45 ± 1.29	−1.50 ± 1.19	−1.38 ± 1.46	**< 0.01**	**0.02** a
Shwachman–Kulczycki score	77.5 (40‐90)	57.5 (40‐90)	65 (40–90)	55 (40–85)	< **0.01** #	< **0.01** a ^**‐**^ b, *
Genotype, %						
F508del homozygous	9 (18)	3 (8.3)	1 (4.7)	2 (13.3)	0.31	0.55
F508del heterozygous	16 (32)	16 (44.4)	10 (47.6)	6 (40)		
Others	25 (50)	17 (47.2)	10 (47.6)	7 (46.6)		
CF‐related disease, (%)						
Pancreatic insufficiency	46 (92)	34 (94.4)	20 (95.2)	14 (93.3)	0.50	0.87
Liver disease	4 (8)	7 (19.4)	4 (19)	3 (20)	0.10	0.29
Diabetes	4 (8)	8 (25)	4 (19)	4 (26.6)	0.06	0.13
Colonization, %	30 (60)	29 (80.5)	17 (80.9)	12 (80)	**0.03**	0.12
*Staphylococcus aureus*	16 (32)	11 (30.5)	5 (23.8)	6 (40)	0.53	0.57
*Pseudomonas aeruginosa*	19 (38)	20 (55.5)	13 (61.9)	7 (46.6)	0.08	0.18
Fungal	12 (24)	11 (30.5)	5 (23.8)	6 (40)	0.33	0.44
Nutritional status						
Gastrostomy tube	1 (2)	3 (8.3)	2 (9.5)	1 (6.6)	0.19	0.35
vitamin/mineral supplementation	49 (98)	36 (100)	21 (100)	15 (100)	0.85	0.65
Medical treatment						
Systemic steroid	1 (2)	4 (11.1)	1 (4.7)	3 (20)	0.09	**0.03** b
Inhaled steroid	7 (14)	9 (25)	4 (19)	5 (33.3)	0.15	0.24
Acid suppression	9 (18)	12 (33.3)	6 (28.5)	6 (40)	0.08	0.19
Annual pulmonary exacerbations	1 (0–4)	1 (0–4)	1 (0–4)	1 (0–4)	**0.03**	**0.01** a ^**‐**^ b
History of fracture	—	1 (2.7)	—	1 (6.6)	—	—
Respiratory support	2 (4)	8 (22.2)	5 (23.8)	3 (20)	**< 0.01**	**0.05** a ^**‐**^ b
Evaluation for LTx	4 (8)	12 (33.3)	7 (33.3)	5 (33.3)	< **0.01**	< **0.05** a ^**‐**^ b
Physical activity, (%)					0.97	0.68
No activity	31 (62)	22 (61.1)	12 (57.1)	10 (66.6)		
1–3 days/week	16 (32)	12 (33.3)	7 (33.3)	5 (33.3)		
≥ 3 days/week	3 (6)	2 (5.5)	2 (9.5)	—		
Spirometry						
FEV_1_	−1.68 ± 1.99	−2.73 ± 2.47	−2.66 ± 2.70	−2.82 ± 2.24	**0.04**	0.12
FVC	−2.08 ± 1.88	−3.19 ± 2.16	−3.20 ± 2.50	−3.17 ± 2.27	**0.02**	0.08
FEV_1_/FVC	0.68 ± 1.44	0.55 ± 1.52	0.68 ± 1.50	0.39 ± 1.59	0.71	0.80
FEF_25–75_	−0.61 ± 2.01	−1.27 ± 2.70	−1.09 ± 2.53	−1.51 ± 3.01	0.23	0.43
Laboratory data						
Biochemical data						
Fasting blood sugar, mg/dL	86.16 ± 10.66	89.40 ± 12.81	89.14 ± 13.02	89.78 ± 12.98	0.20	0.45
Albumin, gr/L	44 (36–49)	41.8 (33–49.8)	41.8 (33–49.8)	41.5 (35–48)	**< 0.01** #	**0.01** a, *
Systemic inflammation markers						
*c*‐reactive protein, mg/L	0.35 (0–3.99)	1.90 (0.00–5.94)	1.40 (0–5.94)	1.90 (0.03–5.60)	< **0.01** #	< **0.01** a ^**‐**^ b, *
Immunoglobulin G, mg/dL	1276.55 ± 339.40	1405.57 ± 539.96	1365.50 ± 551.60	1445.81 ± 553.08	0.27	0.50
Bone metabolism‐related markers						
Calcium, mg/dL	9.59 ± 0.42	9.50 ± 0.39	9.53 ± 0.41	9.45 ± 0.36	0.31	0.53
Phosphorus, mg/dL	4.54 ± 0.53	4.55 ± 0.71	4.58 ± 0.80	4.49 ± 0.56	0.98	0.90
Magnesium, mg/dL	2.08 ± 0.22	2.04 ± 0.25	2.03 ± 0.31	2.06 ± 0.12	0.42	0.68
Alkaline phosphate, U/L	232.86 ± 109.77	197.80 ± 71.17	188.33 ± 77.99	212.00 ± 59.39	0.10	0.20
Vitamin D level, ng/mL	32.34 ± 14.41	32.08 ± 20.37	27.28 ± 11.03	39.28 ± 28.38	0.94	0.12
Parathyroid hormone, ng/L	39.61 ± 16.63	37.60 ± 18.26	38.76 ± 22.13	35.82 ± 10.56	0.64	0.81
Osteocalcin, µg/L	61.10 ± 44.62	55.47 ± 36.95	54.27 ± 30.41	57.54 ± 48.92	0.66	0.90

^1^ Comparison between BMD > −1 and BMD < −1.

^2^ Comparison between normal, moderately low, and very low.

^a^ Comparison between normal and moderately low.

^b^ Comparison between normal and very low.

^c^ Comparison between moderately low and very low.

^#^ Mann–Whitney *U* test.

* Kruskal–Wallis test.

Body mass index *z* score was lower in the group with abnormal BMD compared to normal BMD (*p* < 0.01), the statistically significant difference was only between normal and moderately low BMD (*p*: 0.02). Patients were categorized as having BMI < 50th percentile and BMI ≥ 50th percentile. 68% of children in the normal BMD have a BMI < 50th percentile and 86.1% of children in the abnormal BMD (90.4% in moderately low and 80% in very low BMD). There was a statistically significant difference between the normal and abnormal BMD and the normal and moderately low groups (*p* : 0.04).

The Shwachman–Kulczycki score was lower in the abnormal BMD than in the normal group (57.5 vs 77.5, *p* < 0.01). When the scores were grouped according to points, below good clinical scores (≤ 70 points), a statistically significant difference was found between the normal and moderately low BMD (30% vs 75%, *p* < 0.01), and normal and very low groups (30% vs 80%, *p* < 0.05).

Microorganism colonization and the number of annual pulmonary exacerbations were frequent in the abnormal BMD group compared to normal BMD [(80% vs 60%, *p* : 0.03) and (*p* < 0.03)]. However, the frequency of colonization with specific pathogens such as *S. aureus and P. aeruginosa* was also similar between the groups (*p* > 0.05). Annual pulmonary exacerbation frequencies were grouped as < 2/year and ≥ 2/year. In the patients with ≥ 2 annual exacerbations, the statistically significant differences were in the groups with both normal and moderately low, and normal and very low BMD (*p* < 0.05). In the normal BMD group, 12% of patients had ≥ 2 pulmonary exacerbations per year, 47.6% in the moderately low, and 46.6% in the very low BMD.

Patients with respiratory support and evaluation for LTx were frequently in abnormal BMD (22.2% and 33.3%), both moderately low (23.8% and 33.3%, *p* < 0.05) and very low BMD (20% and 33.3%, *p* < 0.05), compared to normal BMD (4% and 8%).

#### 3.1.2 PFTs

FEV_1_ and FVC *z* scores were lower in abnormal compared to normal BMD (−1.68 ± 1.99 vs −2.73 ± 2.47, *p* : 0.04 and −2.08 ± 1.88 vs −3.19 ± 2.16, *p*: 0.02). PFTs are divided into normal and low according to the FEV1 *z* score. PTFs were low in 48% of children in the normal BMD, 61.9% in the moderately low BMD, and 80% in the very low BMD, and a statistically significant difference was in normal and moderately low, and normal and very low groups (*p* < 0.05).

#### 3.1.3 Laboratory Data

Among the laboratory data, only albumin and CRP levels showed statistically significant differences between the groups (normal vs moderately low and normal vs very low BMD). Median albumin levels were higher and median CRP levels were lower in normal BMD (44 gr/L and 0.35 mg/L) compared to moderately low (41.8 gr/L and 1.40 mg/L) and very low BMD (41.5 gr/L and 1.90 mg/L) groups (*p* < 0.05) (Table 3).

**Table 3:** Comparison of risk factors in BMD groups.

	BMD > −1	BMD < −1				
	Normal (*N* = 50)	Total (*N* = 36)	BMD −1 and −2 Moderately low (*N* = 21)	BMD < −2 Very low (*N* = 15)	p^1^	p^2^
Age groups					0.99	0.49
6–8 years	11 (22)	8 (22.2)	5 (23.8)	3 (20)		
8–10 years	14 (28)	10 (27.7)	8 (38)	2 (13.3)		
> 10 years	25 (50)	18 (50)	8 (38)	10 (66.6)		
BMI, %						
< 50th percentile	34 (68)	31 (86.1)	19 (90.4)	12 (80)	**0.04**	**0.04** a
≥ 50th percentile	16 (32)	5 (13.8)	2 (9.5)	3 (20)		
Shwachman–Kulczycki score						
> 85/ ≤ 85	7/43	2/34	2/19	−/15	0.18	0.29
> 70/ ≤ 70	35/15	9/27	6/15	3/12	**< 0.01**	**< 0.05** a ^**‐**^ b
Colonization, %	30 (60)	29 (80.5)	17 (80.9)	12 (80)	**0.03**	0.12
Annual pulmonary exacerbations						
< 2 and ≥ 2/year	44/6	19/17	11/10	8/7	< **0.01**	**< 0.05** a ^**‐**^ b
Respiratory support, %	2 (4)	8 (22.2)	5 (23.8)	3 (20)	< **0.01**	< **0.05** a ^**‐**^ b
Evaluation for LTx, %	4 (8)	12 (33.3)	7 (33.3)	5 (33.3)	< **0.01**	< **0.05** a ^**‐**^ b
Spirometry, %						
FEV_1_						
> −1.65 (normal)	26 (52)	11 (30.5)	8 (38)	3 (20)	**0.03**	**< 0.05** b
< −1.65 (low)	24 (48)	25 (69.4)	13 (61.9)	12 (80)		
Laboratory data						
Biochemical data						
Albumin, gr/L	44 (36–49)	41.8 (33–49.8)	41.8 (33–49.8)	41.5 (35–48)	**< 0.01** #	**0.01** a, *
Systemic inflammation markers						
*c*‐reactive protein, mg/L	0.35 (0–3.99)	1.90 (0.00–5.94)	1.40 (0–5.94)	1.90 (0.03–5.60)	< **0.01** #	< **0.05** a ^**‐**^ b, *

^1^ Comparison between BMD > −1 and BMD < −1.

^2^ Comparison between normal, moderately low, and very low.

^a^ Comparison between normal and moderately low.

^b^ Comparison between normal and very low.

^c^ Comparison between moderately low and very low.

^#^ Mann–Whitney U test.

* Kruskal–Wallis test.

#### 3.1.4 Correlation Between BMD *Z* Scores and Clinical Measurements

There was a significant positive correlation between BMD *z*‐score and BMI *z*‐score (*r* : 0.30 and *p* < 0.01) and SKS (*r* : 0.36 and *p* < 0.01) in demographic and clinical measurements and FEV_1_ *z*‐score (*r* : 0.25 and *p*: 0.02) in PFTs. In laboratory data, there was a significant positive correlation between BMD *z* score and albumin (*r* : 0.28 and *p* < 0.01) and a significant negative correlation with CRP (*r* : −0.34 and *p* < 0.01).

Considering the significant differences detected between normal and abnormal BMD groups, logistic regression analysis was performed to determine the predictable risk factor for low BMD. CRP increase was found to be significant in predicting low BMD (Table 4).

**Table 4:** Logistic regression analysis to determine the predictable risk factor for low BMD.

Variable	*β*	OR	*p*	95% CI
BMI	0.52	1.69	0.43	0.44–6.43
SKS	1.16	3.22	0.11	0.74–13.93
Colonization	0.20	1.23	0.75	0.34–4.43
Annual pulmonary exacerbations	0.96	2.61	0.23	0.53–12.87
Respiratory support	0.04	1.04	0.96	0.13–8.26
FEV_1_	−1.32	0.26	0.09	0.05–1.25
CRP	0.64	1.89	**0.03**	1.05–3.40
Albumin	−0.90	0.40	0.31	0.07–2.34

*Note:* BMI; < 50th percentile, SKS; ≤ 70 points, colonization; the presence of respiratory microorganism colonization, Annual pulmonary exacerbations; ≥ 2 exacerbation per year, FEV1 < −1.65, adjusted R2 = 0.39. β; beta‐the estimated coefficient, OR; odds ratio, 95% CI; the 95% confidence interval. Bold value is statistically significant.

## 4 Discussion

This study reflects pediatric LTx center data on CFBD, which has become a prominent complication due to increased life expectancy with new developments and modulatory therapies. The rate of DXA screening in our center was 81.1% over 8 years of age and 72.8% over 6 years of age. 41.8% of our population had abnormal BMD, and the rate of very low BMD was 17.4%. The frequency of abnormal BMD was higher in children with BMI < 50th percentile, SKS ≤ 70, low FEV_1_ *z* score, respiratory microorganism colonization, ≥ 2 annual pulmonary exacerbations, required respiratory support, low albumin, and high CRP levels. Systemic inflammation marker CRP increase was the most predictable parameter for low BMD.

The decrease in BMD in CF may be clinically silent, and there is no specific clinical or laboratory indicator to raise suspicion. Early recognition of this condition in children is important, especially in patients with risk factors and poor clinical conditions. Importantly, our results suggest that even patients with moderately low BMD, besides those with very low BMD, are associated with several risk factors. This indicates that patients in this group should also be carefully monitored and prioritized in clinical management strategies. As a transplant center, the management and treatment of very low BMD patients may cause disruptions and delays in transplant evaluation processes. For this reason, we aimed to take the necessary measures to eliminate the disruptions seen in the transition to adulthood or transplantation processes, starting from managing patients with moderately low BMD.

Studies have reported that the prevalence of moderately low BMD was 28‐40% and very low BMD was 9%–17% in children with CF [13, 14, 15]. In this study, the prevalence of moderately low BMD and very low BMD was 24% and 17%, respectively, consistent with the literature. There are studies on screening rates of DXA scans in adult CF patients, but very few were on pediatric patients. In a single‐center retrospective study in Australia between 2000 and 2016 the rate of patients who underwent at least one DXA scan was 45% [16]. In contrast to this study and inadequate screening rates in adults, our study's relatively high DXA scan rates may be due to being an LTx center with a high awareness of CFBD. The factors affecting our screening rate were evaluated and the patient's clinical status and regular follow‐up were the most important determinants of screening. The screening was postponed to older ages in children with good clinical status and became of secondary importance because other problems were addressed during visits in children without regular follow‐ups. An action plan was created to ensure the continuity of regular follow‐up of patients. Children with a lack of clinical data, without DXA scans and regular follow‐ups, were invited to the clinic and their examinations and tests were completed. Screening information regarding CFBD of five children being followed up at a different center was given to the centers. Ten children aged 6–8 years with good clinical condition were followed up for risk factors.

The pathophysiology of low BMD in CF is multifactorial and determining predictive causes is difficult due to different clinical outcomes. Current guidelines recommend that screening with DXA should start at the age of 8 years, as bone loss is most common in the peripubertal age [3, 17]. However, studies suggest that screening can be performed at an earlier age in children with known risk factors for low BMD. In our study, approximately 22% of the study population consisted of patients in the 6‐8 age group and 42.1% of them had abnormal BMD. The reason for the DXA scan performed in this age group was poor clinical status and risk factors. BMD z scores were similar in age groups. Also similarly, in a study by Sermet‐Gaudelus et al. in young children, BMD z scores were similar in the age groups < 6 years (*N*: 25), 6–10 years (*N*: 53), and 11–18 years (*N*: 36). Although most of the younger than 6 years children had normal nutritional status and mild pulmonary disease, 34% of patients had abnormal BMD [18].

The study found no statistically significant difference between BMD groups in terms of known risk factors such as CFTR dysfunction, physical activity, pancreatic insufficiency, vitamin and mineral deficiencies, and CFDM. One reason for this is our study group's high genetic diversity. Chadwick et al. examined DXA results of 141 pediatric patients along with concomitant diseases, genetics, anthropometric measurements, drug exposure, and relevant serum studies and found that most patients had at least one ΔF508 mutation (79.6%) with a higher percentage of children with abnormal DXA [19]. Unlike countries where the ΔF508 mutation is common, studies about the effects of CFTR dysfunction on bone disease in countries with high genetic diversity, such as ours, may provide detailed information on the function and impact of the CFTR protein.

Another contributing factor to our findings was that the majority of the study population consisted of children with pancreatic insufficiency who were receiving vitamin and mineral supplementation. Despite similar rates of pancreatic insufficiency and supplementation across groups, BMI *z*‐scores were significantly lower in the abnormal BMD group compared to the normal BMD group. This disparity may be explained by differences in nutrient intake, as well as the impact of chronic inflammation on growth and nutritional status. Over the years, accumulating evidence in CF has highlighted the strong relationship between nutritional status and disease‐related comorbidities. However, data from patient registries and previous studies indicate that achieving and maintaining optimal nutritional status remains challenging in many cases. When we stratified patients by BMI percentiles, a statistically significant difference emerged between groups below the 50th percentile. This finding aligns with current guidelines, which recommend that children with CF aged 2–18 years maintain a BMI between the 50th and 85th percentiles [20]. We obtained two important conclusions from this result: (1) to be careful in terms of loss of BMD in children with BMI < 50th percentile, and (2) that an action plan should be created regarding the current nutritional status of the patients in our cohort. Following a multidisciplinary assessment of factors contributing to weight loss or insufficient weight gain (e.g., pancreatic enzyme replacement therapy inadequacy, celiac disease), tailored nutrition plans were designed in collaboration with dietitians, with specific attention to the BMD classification (moderately low vs very low). Furthermore, routine dietitian evaluations were incorporated into the follow‐up protocol during periods of pulmonary exacerbation. Although no significant differences were observed between BMD groups in terms of vitamin and mineral deficiencies (e.g., vitamin D, calcium), the supplementation needs of all patients were reassessed as part of comprehensive nutritional management.

Chronic inflammation and recurrent pulmonary exacerbations induce pro‐inflammatory cytokine production, cause a catabolic state, and are strongly associated with BMD [4, 16, 19, 21, 22]. In a study on 125 adult CF patients, a significant and independent association between femoral BMD *z* score and increased high sensitive CRP levels in patients with very low BMD [23]. The higher CRP levels, respiratory microorganism colonization, ≥ 2 annual pulmonary exacerbations, and low albumin with negative acute phase reactant property in the abnormal BMD group in our study may support the relationship between inflammation and bone loss. In our unit, we have identified these criteria with BMI < 50th percentile, SKS ≤ 70, low PTFs, and albumin levels as risk factors for bone loss to be considered and reviewed.

One of the most significant deficiencies identified in the study was that 60% of the study population did not engage in regular physical activity, despite undergoing annual physical therapy evaluations. According to the Cochrane review, physical exercise is an integral component of multidisciplinary care, and current guidelines recommend incorporating high‐impact weight‐bearing exercises for 20–30 min, three times a week, in addition to routine daily activities. However, there is still a limited number of studies demonstrating a direct benefit of such interventions on clinical outcomes in children with cystic fibrosis [3, 24]. In a study conducted by Jantzen et al. involving both pediatric and adult patients with cystic fibrosis (CF), overall physical activity levels in individuals with CF were similar to healthy controls, and beside this, young school‐age children (6–13 years) were less likely to participate in strenuous activities than their healthy peers [25]. In our unit, an intensive physical therapy assessment and exercise program is already implemented as part of the pre‐transplantation protocol. Nevertheless, to address this current shortcoming, we plan to develop a tailored physical therapy program stratified according to the BMD groups of the children and to ensure regular monitoring of adherence and progress.

The most important limitation of our study was a retrospective study. However, this study was designed to provide an overview of our center and to improve our follow‐up for CFBD. Another limitation was that the puberty status of the children could not be routinely recorded in clinic visits, and also examined as a predisposing factor for bone disease, since 50% of the study population was below 10 years of age. The effects of modulatory therapy on bone health and treatment protocols for patients with CFBD were not addressed in this study, as this is a topic for a separate evaluation. The study primarily uses cross‐sectional data from DXA scans at single time points. Longitudinal data tracking bone density changes over time would provide more insight into the progression of CFBD and the impact of interventions.

**Figure 2:**
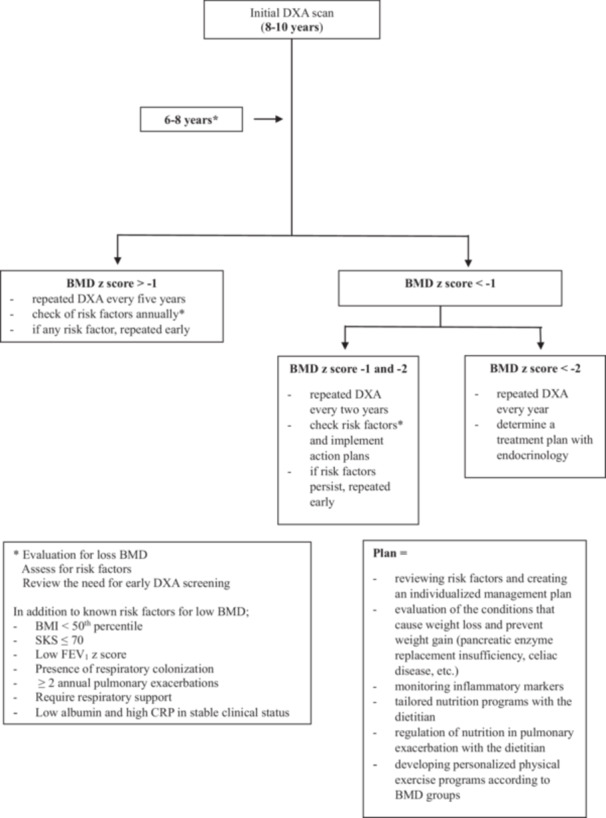
Algorithm for improving our CFBD screening program.

In conclusion, our study highlights that key clinical indicators such as low BMI, poor clinical scores (SKS ≤ 70), impaired lung function, systemic inflammation (elevated CRP), and nutritional deficiencies were significantly associated with reduced BMD. Importantly, children with moderately low BMD also exhibited these risk factors, underscoring the need for early intervention—not only for those with very low BMD. Our findings emphasize the importance of a proactive, multidisciplinary approach that includes routine DXA screening, individualized nutrition strategies, consistent physical therapy engagement, and risk‐based monitoring starting as early as age 6 for selected patients. We created new strategic plans to improve our CFBD screening program by evaluating our results as a “Heart‐Lung Transplant Unit” in the literature and guidelines recommendations (Figure 2). These measures can help optimize bone health, improve long‐term outcomes, quality of life, and better prepare children with CF for potential future transplantation.

## Author Contributions

**Gökçen Kartal Öztürk:** conceptualization, investigation, writing – original draft, writing – review and editing, project administration, supervision, methodology, validation, software, formal analysis, data curation, resources. **Ece Halis:** data curation, software, formal analysis, writing – review and editing. **Ece Ocak:** conceptualization, investigation, writing – review and editing, visualization, methodology, software. **Aykut Eşki:** software, formal analysis, data curation, methodology, investigation. **Damla Gökşen:** conceptualization, investigation, writing – original draft, writing – review and editing, methodology. **Samim Özen:** conceptualization, investigation, writing – original draft, methodology, writing – review and editing. **Fevziye Çoksüer:** software, formal analysis, data curation. **Esen Demir:** conceptualization, investigation, writing – original draft, writing – review and editing, methodology. **Figen Gülen:** conceptualization, investigation, writing – original draft, methodology, writing – review and editing.

## Ethics Statement

The Local Research Ethics Committee (20‐6 T/56) approved this study, which was conducted in accordance with the principles outlined in the Declaration of Helsinki.

## Consent to Participate

Informed consent was obtained from all subjects and their parents.

## Consent for Publication

The authors have nothing to report.

## Conflicts of Interest

The authors declare no conflicts of interest.

## Data Availability

The data that support the findings of this study are available from the corresponding author upon reasonable request.
